# Linear Response Equations Revisited: A Simple and Efficient Iterative Algorithm

**DOI:** 10.1021/acs.jctc.3c00989

**Published:** 2023-12-11

**Authors:** Riccardo Alessandro, Ivan Giannì, Federica Pes, Tommaso Nottoli, Filippo Lipparini

**Affiliations:** Dipartimento di Chimica e Chimica Industriale, Università di Pisa Via G. Moruzzi 13, 56124 Pisa, Italy

## Abstract

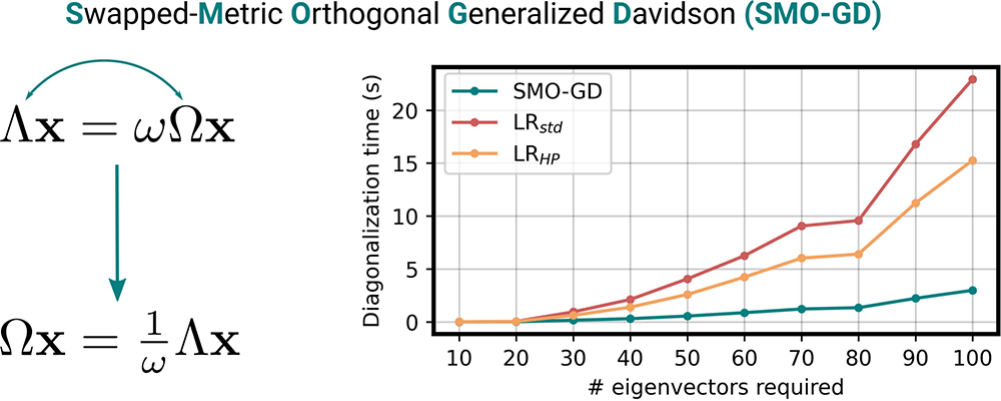

We present an algorithm to solve the linear response equations for Hartree–Fock, Density Functional Theory, and the Multiconfigurational Self-Consistent Field method that is both simple and efficient. The algorithm makes use of the well-established symmetric and antisymmetric combinations of trial vectors but further orthogonalizes them with respect to the scalar product induced by the response matrix. This leads to a standard, symmetric block eigenvalue problem in the expansion subspace that can be solved by diagonalizing a symmetric, positive definite matrix half the size of the expansion space. Numerical tests showed that the algorithm is robust and stable.

Linear response calculations^1^ are commonly encountered in computational chemistry, because they are used to compute excitation energies and transition properties. Such calculations are available at virtually any level of theory, from Hartree–Fock (HF) and Kohn–Sham Density Functional Theory (KS-DFT),^2−4^ to coupled-cluster theory,^5−8^ to Multiconfigurational Self-Consistent Field (MCSCF),^7,9−11^ just to name a few. Given the importance and usefulness of such calculations, algorithms that allow for efficient implementation are paramount. In this short communication, we focus on self-consistent field (SCF) methods, i.e., HF, density functional theory (DFT), and MCSCF. In particular, we present the equations and detail the algorithm for MCSCF, as HF and DFT can be considered as special cases of the former.

The linear response equations for MCSCF have the general form, assuming that real basis functions are used:^1,7,9−13^
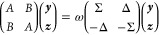
1where *A*, *B*, and Σ are symmetric matrices in , Δ is an antisymmetric matrix in , and ***y***, ***z*** are vectors in . Here, *n* represents the size of the problem, e.g., the number of occupied times number of virtual orbitals for HF and KS-DFT, or the number of orbital rotations and independent CI parameters for MCSCF. We further assume that Σ is positive definite, as are the combinations *A* + *B* and *A* – *B*, conditions that are met if the ground-state solution is stable. The eigenvalue problem (eq 1) has been investigated in the standard form as well as in the generalized one: in refs (14−16), the authors developed a minimization principle to find a few smallest eigenvalues and the corresponding eigenvectors. For HF and KS-DFT response theory, eq 1 is simplified, as Σ becomes the identity matrix and Δ becomes the zero matrix. For the sake of brevity, we also write the response equations as
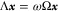
2eq 2 is a generalized eigenvalue problem with a nonpositive-definite metric Ω, which means that the standard procedure used in other quantum chemical calculations, where either a Cholesky decomposition of the metric or Ω^–1/2^ are computed in order to transform eq 2 into a standard eigenvalue problem, cannot be used. The various algorithms that have been proposed to tackle such a problem must, therefore, deal with this complication. A few of the main strategies proposed in the literature are discussed in the following.

The linear response equations have an important property. If {ω, (***y***, ***z***)^T^} is a solution, then {−ω, (***z***, ***y***)^T^} is also a solution to the same problem. In other words, the eigenvalues of eq 2 always appear as positive–negative pairs. It has been suggested in the literature^17−20^ that this symmetry should be encoded in the iterative algorithm used to solve the response equations, usually a modified version of Davidson’s method,^21^ by expanding each eigenvector ***x*** as the linear combination of two sets of vectors:
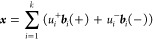
3where  and the expansion vectors are defined as follows:
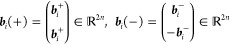
4with . In the following, we use the notation, for a generic vector , ***v***(+) = (***v***^+^, ***v***^+^)^*T*^ and ***v***(−) = (***v***^–^, – ***v***^–^)^*T*^, with  to denote symmetric and antisymmetric vectors, respectively. Furthermore, to keep the notation simple, we present the derivation of the equations for the case where only one eigenpair is sought: the generalization to many eigenpairs is straightforward. This choice of expansion spaces is particularly advantageous, as it gives rise to blocked reduced matrices for problem 1. In fact,
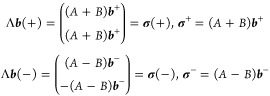
5and
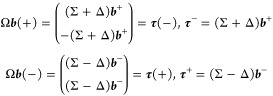
6In the spirit of Davidson’s method, the response equations can be therefore solved as follows.

Let  and  be the symmetric and antisymmetric expansion subspaces, respectively, whose dimension increases with *k*. Here *k* represents the number of iterations already performed. Let
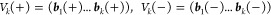
be matrices in  where the columns are the symmetric and antisymmetric expansion vectors, respectively. Let the columns of the matrices *V*_*k*_(±) form an orthonormal basis for the subspaces . Let us also introduce the spaces
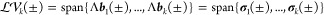
and
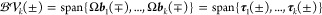
that collect the applications of the matrices Λ and Ω to the expansion vectors. We remark that, in practice, only the vectors  are stored in memory. Specifically, we consider the *n* × *k* matrices *V*_*k*_^±^ = (***b***_1_^±^...***b***_*k*_^±^), the spaces  and , and the corresponding matrices *LV*_*k*_^±^ = (**σ**_1_^±^...**σ**_*k*_^±^) and *BV*_*k*_^±^ = (**τ**_1_^±^...**τ**_*k*_^±^).

The Rayleigh–Ritz variational procedure is then performed to compute the ***u***^±^ coefficients from eq 3 by solving the projection of eq 1 into the subspace union of *V*_*k*_^+^ and *V*_*k*_^–^. We get the 2*k* × 2*k* problem
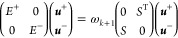
7where
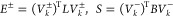
8that is, for *i* = 1, ...,*k* and *j* = 1, ...,*i*,
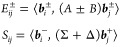
We note here that eq 7 has the same structure of the original response equation, with a nonpositive definite metric. However, assuming that ω ≠ 0, it is possible to divide both sides of the reduced equation by ω obtaining
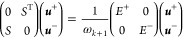
9where now the reduced response matrix, which is symmetric and positive definite, acts as a metric, making it possible to use standard linear algebra techniques.

Once the eigenvalue ω_*k*+1_ is computed in the reduced space and the current approximation to the eigenvector ***x***_*k*+1_ has been computed as in eq 3,
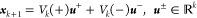
10we can assemble the residual vector *R*_*k*+1_ in the full space 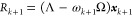
11which emerges naturally as the sum of a symmetric term *R*_*k*+1_(+) and an antisymmetric term *R*_*k*+1_(−), whose components are expressed in terms of elements of the spaces *LV*_*k*_^±^ and *BV*_*k*_^±^, i.e.,
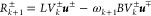
12

The expansion subspaces are extended, in the next iteration, using the standard Davidson algorithm, i.e., by solving
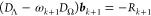
13where *D*_Λ_ and *D*_Ω_ are diagonal matrices whose elements are the diagonal elements of Λ and Ω, respectively, and ***b***_*k*+1_ = ***b***_*k*+1_(+) + ***b***_*k*+1_(−). Splitting eq 13 into a symmetric and an antisymmetric part we get, through simple algebra,
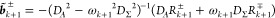
14where *D*_*A*_ and *D*_Σ_ are the diagonals of *A* and Σ, respectively. The ***b*~**_*k*+1_^±^ vectors are then orthogonalized to *V*_*k*_^±^, respectively, and the procedure is iterated until convergence is reached. Each iteration requires four matrix-vector products (MVP), to compute **σ**_*k*_^+^, **σ**_*k*_^–^, **τ**_*k*_^+^, and **τ**_*k*_^–^, where the first two MVP are typically the cost-dominating step (see eqs 5 and 6). However, the cost of solving the generalized eigenvalue problem (eq 7) and the cost of orthogonalizing the new expansion vectors can also become computational bottlenecks, especially if a large number *n*_eig_ of eigenvalues and eigenvectors are sought. To lower the cost of the former operation, Helmich-Paris^20^ recently proposed a method to reduce the generalized eigenvalue problem into a half-dimensional problem, i.e., the size of one of the blocks *E*^±^ and of *S*. The proposed algorithm scales with , where *m* = *k* · *n*_eig_ is the size of the subspace, which is an important improvement with respect to the  solution to eq 7. However, it requires a sizable number of linear algebra operations, including two singular value decompositions (SVD), which can become rather expensive.

In this paper, we propose a different approach that results in a much simpler implementation and that performs, in a *m*-dimensional space, only one diagonalization and one matrix–matrix multiplication. Here, we only consider the case where a single eigenpair is searched, i.e., *m* = *k*.

We start by noting that, if the ground-state solution is stable, problem (2) has no vanishing eigenvalue, and, therefore, it is possible to rewrite it as^22^
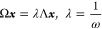
15Problem (15) is much easier to solve than problem (2), because it is a generalized eigenvalue problem with a symmetric, positive definite metric Λ. It is therefore possible to introduce a positive definite dot product
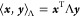
16and the induced Λ-norm . We proceed similarly to what has already been presented and expand the eigenvector ***x*** as the linear combination of symmetric and antisymmetric expansion vectors as in eq 3, where, in our approach, we choose the expansion vectors such that
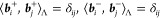
17that is, the expansion vectors are Λ-orthogonal.

With the definition of *S* given in eq 8, we obtain the following reduced problem:
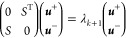
18which, due to the choice of Λ-orthogonal expansion vectors, is no longer a generalized eigenvalue problem. Furthermore, its block structure allows us to solve it by first computing ***u***^+^ from
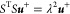
19a symmetric and positive definite eigenvalue problem of size *m*, and then by recovering ***u***^–^ as
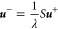
20After assembling the current approximation to the eigenvector, we compute the residual
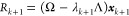
21which can be split into a symmetric and an antisymmetric term, whose components are
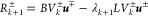
22The new pair of expansion vectors are then obtained by solving
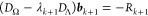
23Splitting the new vector and the residual in the sum of symmetric and antisymmetric components, after some computation, we get
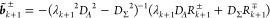
24Once the ***b*~**_*k*+1_^±^ vectors have been computed, we enlarge the expansion subspaces  by determining vectors that are orthogonal to *V*_*k*_^±^. To this end, the new vectors ***b*~**_*k*+1_^±^ are first orthogonalized to *V*_*k*_^±^ and then orthonormalized. This is done as follows: first, we project out *V*_*k*_^±^ from the vectors
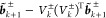
25and then we orthonormalize the resulting vectors using the Cholesky decomposition of their overlap, as described in a recent paper by some of us.^23^ To ensure the numerical stability of this procedure, these two steps are iterated until the norm of (*V*_*k*_^±^)^T^***b*~**_*k*+1_^±^ is smaller than a (tight) threshold. In all of our numerical experiments, two iterations were sufficient to obtain vectors orthogonal to machine precision. The resulting vectors ***b*^**_*k*+1_^±^ are then orthogonalized with respect to Λ by computing the Cholesky decomposition of
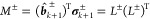
26and then solving the triangular linear system
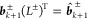
27We remark that, if we are seeking one eigenpair, eq 26 is a trivial case, since *M*^±^ represents a scalar and *L*^±^ is the square root of *M*^±^. This step becomes significant when we want to compute multiple eigenvalues–eigenvectors. Performing the Λ-orthogonalization after regular orthogonalization may look unnecessary, but it vastly improves the conditioning of *M*, thus ensuring the overall numerical stability and robustness of the procedure. Summarizing, first, we compute ***b*~**_*k*+1_^±^; second, we compute ***b*^**_*k*+1_^±^, and finally the expansion vectors ***b***_*k*+1_^±^, which we store on the matrices *V*_*k*+1_^±^ = (*V*_*k*_^±^***b***_*k*+1_^±^). Note that, to perform the latter passage, we must apply *A* + *B* and *A* – *B* to the new test vectors. In other words, the number of MVP in our algorithm is the same as in the traditional ones, but the linear algebra operations are both simpler and less expensive. In particular, the only  operations that we need to perform are the matrix–matrix multiplication *S*^T^*S* and the diagonalization in eq 19. The price to pay is that we need to perform two more orthogonalizations (eqs 26 and 27); however, these have a cost of order , *n*_eig_ being the number of seeked eigenvectors, and should therefore be less critical.
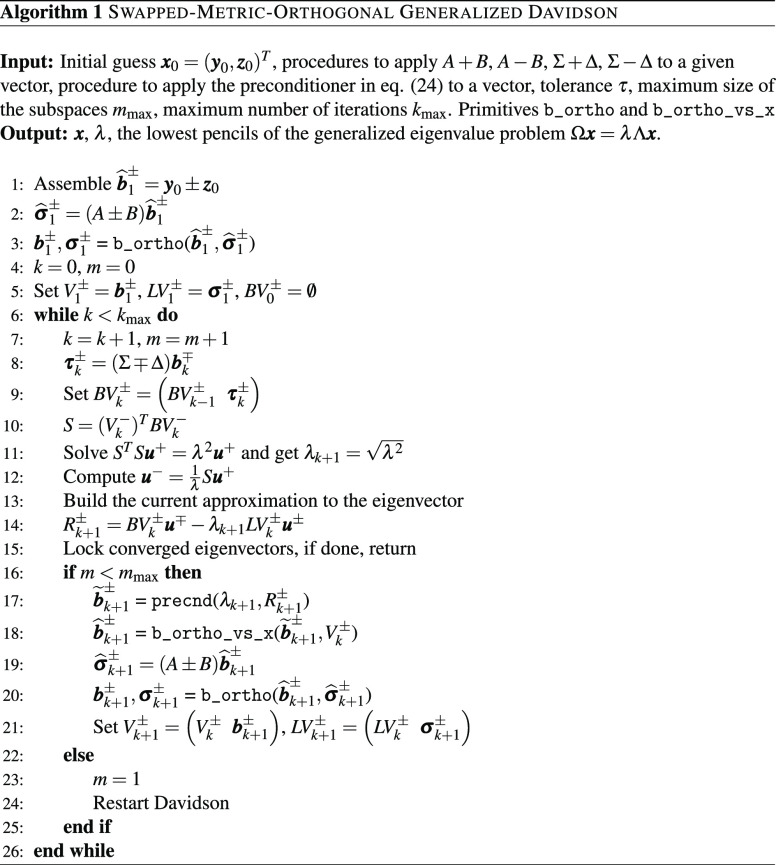


The overall procedure, which we named Swapped-Metric-Orthogonal Generalized Davidson (SMO-GD), is summarized in Algorithm 1. The algorithms of the primitives used for the orthogonalizations are given in the Supporting Information. An open-source (LGPL3), Fortran implementation of the algorithm, including the orthogonalization primitives, can be found in the DiagLib library,^23^ which is available on GitHub at https://github.com/Molecolab-Pisa/diaglib. All of the code used to generate the numerical examples presented in the following can be accessed by downloading the code.

Algorithm 1 is general and can deal with any generalized eigenvalue problem as in eq 1. It exhibits monotonic convergence of the reduced eigenvalues, as the Hylleras–Undheim–McDonald theorem applies,^24,25^ and can be implemented efficiently using Blas and Lapack routines.

In the following, we report a few numerical tests of the proposed algorithm and compare it with the original algorithm proposed by Olsen et al.,^17^ where the 2*m* × 2*m* reduced problem is solved, and the algorithm recently proposed by Helmich-Paris,^20^ which uses a series of transformation to reduce the size of the problem to *m* × *m*. To compare the three algorithms in a fair way, they all have been implemented in the DiagLib library (response branch). As our focus is on the iterative algorithm and not on the implementation of MCSCF (or TD-DFT), we generate test matrices with the correct structure and use in-core matrix–vector multiplications to compute the required matrix–vector products. This allows us not only to validate our implementation against dense Lapack routines but also to create common grounds to compare the three algorithms in a fair way. We focus our analysis on the time required to solve the reduced problem and to orthogonalize the new test vectors against the previous ones, as all of the other steps are identical. We generate symmetric and positive definite *A* + *B* and *A* – *B* matrices by putting
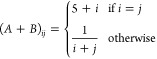
and
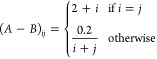
*A* and *B* are then obtained as linear combinations. The symmetric Σ and the antisymmetric Δ matrices are generated in the following way: the former is obtained by generating a random matrix and then multiplying it by its transpose, to ensure that it is symmetric and positive-definite, while the latter antisymmetric matrix is obtained by generating a random matrix and subtracting its transpose.

As a first test example, to investigate the elapsed diagonalization times, we consider a generalized eigenvalue problem of size 2*n* × 2*n*, where *n* = 10 000 is the size of the matrices *A*, *B*, Σ, and Δ. We solved the generalized eigenvalue problem by applying the three algorithms discussed in this work: the original procedure proposed by Olsen and co-workers (LR_std_),^17^ where the reduced problem is solved for 1/ω to have a symmetric, positive-definite metric, the algorithm recently introduced by Helmich-Paris (LR_HP_),^20^ and our implementation shown in Algorithm 1 (SMO-GD). We seek 10–100 eigenpairs with increments of 10. Convergence is achieved when the root-mean-square norm of the residual is smaller than 10^–6^ and its maximum absolute value is smaller than 10^–5^. We use a subspace dimension of 20, i.e., we keep in memory up to 20 vectors per eigenvector in the history. For all of the algorithms, multiple eigenvectors are expanded simultaneously in the same expansion space, and we exploit a locking procedure for the converged eigenvectors. In Figure 1, we report the cumulative elapsed time required to solve the reduced eigenvalue problem for the three algorithms. Such timings are the sum over all the iterations; note that by design, the iterations produced by all algorithms are equivalent, and therefore the number of iterations is shared by all methods. For the algorithm presented here (SMO-GD), the reported timings include the time spent for the additional orthogonalization with respect to the metric (line 20 of Algorithm 1). We observe that the proposed algorithm significantly outperforms both the original Olsen’s algorithm and the new method presented by Helmich-Paris. The differences become more significant as the size of the reduced problem increases, i.e., when more eigenvalues are seeked. Note that in our simple-minded example, the overall cost is still dominated by the dense matrix-vector multiplications. Nevertheless, the difference in timings is sizable and can be expected to have an impact on large-scale applications when many states are computed. To better appreciate the overall difference between the algorithms, we plot, in Figure 2, the total time required to compute 100 eigenvalues and eigenvectors for increasingly larger systems with *n* ranging from 1000 to 10 000. In all cases, our SMO-GD algorithm is faster than the others, and in particular, the time difference increases as the dimension decreases, consistently with what was observed in Figure 1.

**Figure 1:**
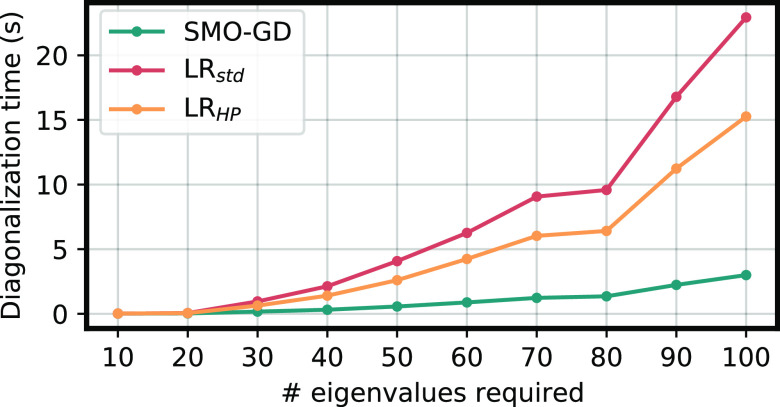
Time analysis of the three methods discussed for a 2*n* × 2*n* problem, where *n* = 10 000. Diagonalization time was calculated with respect to the number of eigenvalues required.

**Figure 2:**
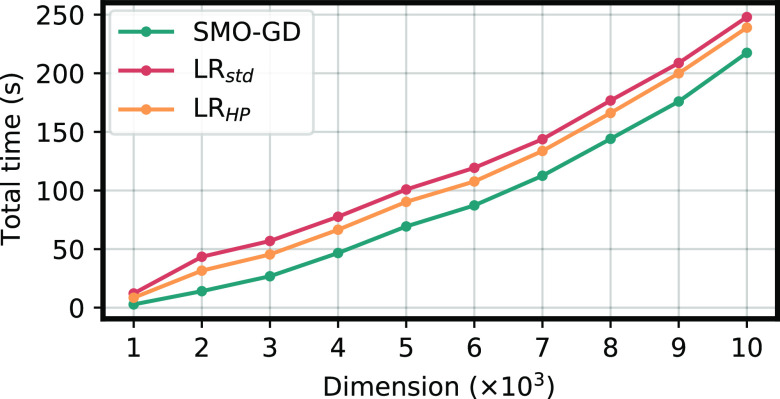
Total time (s) of the three methods for different dimensions *n* and 100 eigenvectors required.

These results can be clearly rationalized by looking at the dense linear algebra operations performed by the various algorithms. Focusing on the leading  operations, the original algorithm by Olsen et al. requires the solution to a generalized eigenvalue problem of size 2*m*, which is performed in our implementation using the DSYGV LAPACK routine, which performs a Cholesky decomposition of the metric to transform the problem into a standard eigenvalue problem and then solves the latter using the DSYEV LAPACK routine. Both operations require  floating-point operations. The algorithm recently presented by Helmich-Paris^20^ avoids  by reducing the subspace problem to a *m*-sized one, by performing two singular value decompositions (LAPACK routine DGESVD), two Cholesky factorizations (LAPACK routine DPOTRF), and eight matrix–matrix multiplications. In contrast, our algorithm requires only one matrix–matrix multiplication (to assemble *S*^*T*^*S* and one symmetric diagonalization (LAPACK routine DSYEV), resulting in a significantly reduced computational cost. The two additional Λ-orthonormalizations require only  operations, which is by definition smaller than *m*.

**Table 1:** Number and Type of  Dense Linear Algebra Operations Required by the Algorithm SMO-GD, Compared with the Strattmann-Scuseria-Frisch (SSF) and Helmich-Paris (HP) Ones^a^

	SMO-GD	SSF	HP
EV	1	2	0
MM	1	5	8
SVD	0	0	2
CD	0	0	2

a Here, *m* is the size of the expansion subspace. EV is the symmetric diagonalization, MM the matrix–matrix multiplication, SVD the singular value decomposition, and CD the Cholesky factorization.

We conclude these remarks by comparing our algorithm to the method proposed by Strattmann, Scuseria, and Frisch (SSF)^4^ for the specific case of LR DFT. The SSF algorithm solves the non-Hermitian problem
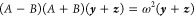
28introducing a nonfaithful representation of eq 28 in the expansion subspace
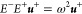
29In their work, they show that the two problems become equivalent when convergence is achieved. Again, the problem in eq 29 is *m*-sized and can be solved by transforming it to the symmetric eigenvalue
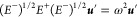
30where ***u***′ = (*E*^–^)^−1/2^***u***^+^. In practice, the implementation requires diagonalization of *E*^–^ to compute (*E*^–^)^1/2^ (one symmetric diagonalization and one matrix–matrix multiplication), which is then used to assemble the symmetric matrix in eq 30 (two matrix–matrix multiplications), which is then diagonalized (a second symmetric diagonalization). Two further matrix–matrix multiplications are used to recover the ***u***^+^ and ***u***^–^ eigenvectors, the latter being the left eigenvectors to eq 29. The left and right eigenvectors ***y*** + ***z*** and ***y*** – ***z*** are then biorthogonalized. A summary of all the dense linear algebra operations performed by the SSF, HP, and SMO-GD algorithms is reported in Table 1. The SSF algorithm generates a different expansion space, with respect to the Olsen algorithm (which generates the same expansion space as the HP algorithm and the one presented in this communication), making a one-to-one comparison somewhat harder, since, in general, the number of iterations may be different. However, results presented by Strattamann, Scuseria, and Frisch in their paper^4^ show that their procedure generates subspaces that are of the same size as the ones generated by the Olsen algorithm, making thus a qualitative comparison possible. We have thus employed both algorithms to solve TD-DFT like equations, obtained by setting Σ = 1 and Δ = 0 in eq 1. We use the same *A* and *B* matrices used in previous tests; however, as these are very well-behaved, starting the iterations from an optimal guess (the canonical basis vectors corresponding to the lowest diagonal elements of *A* + *B*) results in almost immediate convergence of both algorithms and offers little material to compare results. To offer a more realistic comparison, we perturb the optimal guess by adding a random vector to each guess vector, the elements of which are random numbers chosen in the interval [0, 0.01]. The results are reported in Figures 3 and 4. As can be seen from the figure, SMO-GD outperforms SSF in this well-behaved case. The better performances of SMO-GD are due not only to the smaller number of dense linear algebra operations but also to its faster convergence. All the calculations reported in Figures 3 and 4 required in fact 10 SMO-GD and 9 SSF iterations to achieve convergence. As the SSF algorithm performs twice as many matrix-vector multiplications than SMO-GD per iteration, the total number of matrix–vector multiplications is smaller in SMO-GD, which explains the sizable difference in the total elapsed time. We note, in passing, that we have tested the algorithms using randomly generated matrices, where the diagonal was then shifted to make the problem more diagonally dominant and could not achieve convergence using the SSF implementation. On the other hand, SMO-GD always managed to converge. Similar results were obtained by using a very tight (10^–10^ RMS of the residual) convergence threshold. In the latter case, SSF approached convergence and then started to stagnate, while SMO-GD exhibited no problem. Both cases as summarized in the Supporting Information. While neither describes a particularly realistic application scenario, as the response equations in TD-DFT are typically strongly diagonally dominant and thus well-conditioned, we believe that such tests further confirm the good numerical stability of the SMO-GD algorithm.

**Figure 3:**
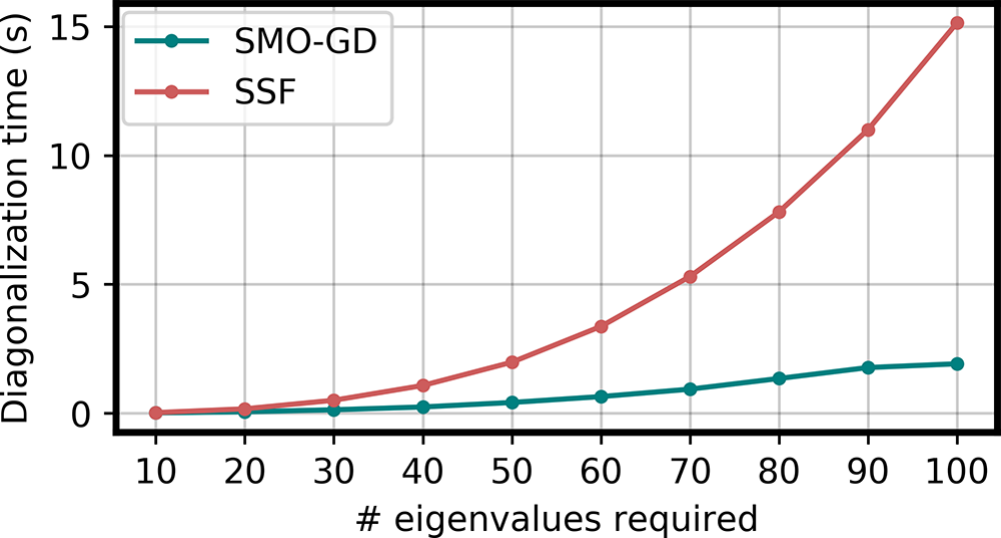
Comparison of the SMO-GD and SSF algorithms for the solution of a 2*n* × 2*n* TD-DFT like generalized eigenvalue equations, with *n* = 10 000 as a function of the number of required eigenvectors. The total time for the dense linear algebra operations required to solve the problem in the subspace is reported for both methods.

**Figure 4:**
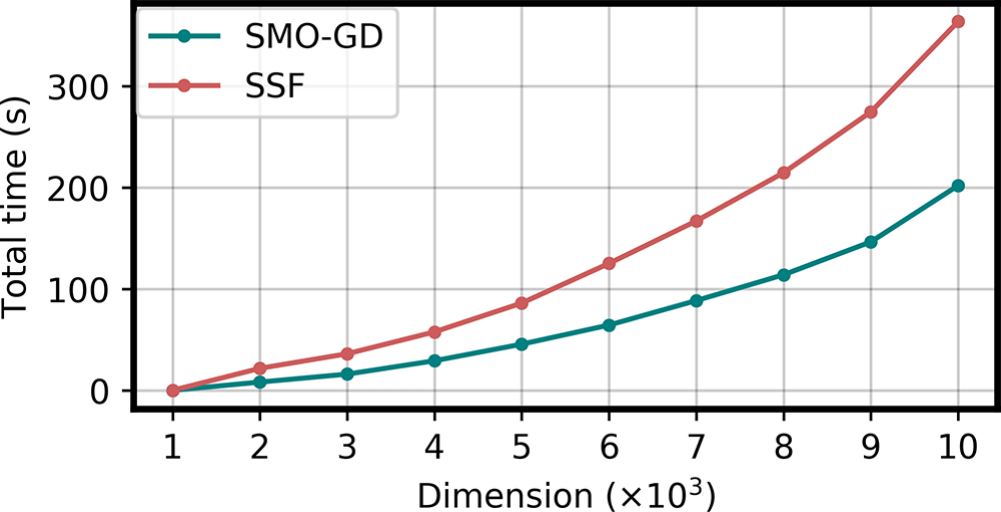
Comparison of the SMO-GD and SSF algorithms for the solution of a 2*n* × 2*n* TD-DFT like generalized eigenvalue equations, with *n* ranging from 1000 to 10 000, when 100 eigenvalues are seeked. The total execution time is reported.

In conclusion, we have presented a new algorithm to solve the MCSCF linear response equations and also the related TD-SCF equations that is not only more efficient than what was previously reported in the literature, but also conceptually simple and easy to implement. Thanks to the robust orthogonalization procedures described in the Supporting Information, it is also numerically robust and stable. If the expansion space becomes ill-conditioned, which is bound to happen near convergence, then the metric in the reduced space can exhibit small (i.e., numerically zero) or even negative eigenvalues, independent of whether the actual metric is ill-conditioned. This can make the overall procedure fail. By choosing expansion vectors that are orthogonal with respect to the scalar product induced by the metric, we avoided this problem from the beginning. Furthermore, by limiting the linear algebra operations to a symmetric diagonalization in the subspace, we avoid further propagation of possible numerical instabilities. The combination of robustness and efficiency makes the new algorithm therefore an ideal strategy to tackle the solution to the linear response equations.

## Supporting Information Available

Pseudocode of the orthogonalization primitives and additional tests and results (PDF)
