# Supplementary material for: Linear Response Equations Revisited: A Simple and Efficient Iterative Algorithm
Source: J Chem Theory Comput. 2023 Dec 11;19(24):9025–31. doi: 10.1021/acs.jctc.3c00989 (PMC10753806; doi:10.1021/acs.jctc.3c00989)
Supplement: Supplementary file 1 — ct3c00989_si_001.pdf [file ct3c00989_si_001.pdf]

Supplemental Material
Linear response equations revisited: a simple and efficient iterative algorithm

Riccardo Alessandro, Ivan Giannì, Federica Pes, Tommaso Nottoli,[*] and Filippo Lipparini[†]
Dipartimento di Chimica e Chimica Industriale, Università di Pisa
Via G. Moruzzi 13, 56124 Pisa, Italy
(Dated: October 30, 2023)

[*] tommaso.nottoli@phd.unipi.it
[†] filippo.lipparini@unipi.it

I. PRIMITIVE ROUTINES

Here, we report the primitive routines deployed in the three algorithms discussed in this work. The orthogonalization of guess trial vectors and new trial vectors with respect to the metric are performed by using routines `b_ortho` and `b_ortho_vs_x` described in Algorithm 1 and Algorithm 2, respectively. Such primitives exploit the routine `ortho_cd` to orthogonalize a set of vectors with iterative refinement as described in Algorithm 3.

Algorithm 1 `b_ortho`$(\widetilde{X}, \widetilde{BX})$: Orthonormalize a set of vectors $\widetilde{X}$ with respect to the metric B using the Cholesky decomposition of the overlap.

Input: non orthogonal vectors $\widetilde{X}$, $\widetilde{BX}$, threshold τ_{ortho}.
Output: X, BX orthonormal vectors.

$M = \widetilde{X}^T \widetilde{BX}$
Cholesky factorization $M = LL^T$
$X = \widetilde{X} L^{-T}$
$BX = \widetilde{BX} L^{-T}$

Algorithm 2 `b_ortho_vs_x`$(X, \widetilde{Y})$: given a set of B-orthonormal vectors X and a set of vectors $\widetilde{Y}$, B-orthogonalize $\widetilde{Y}$ to X and orthonormalize $\overline{Y}$.

Input: B-orthonormal vectors X, non orthogonal vectors $\widetilde{Y}$, threshold τ_{ortho}.
Output: orthogonal vectors $\overline{Y}$, B-orthogonal with respect to X

$Y = \widetilde{Y}$
while $\|Y^T BX\| > \tau_{\text{ortho}}$ **do**
  $Y = Y - X\,(BX)^T\, Y$
  $\overline{Y}$ = `ortho_cd`(Y)
end while

Algorithm 3 `ortho_cd`$(\widetilde{X})$: Orthonormalize a set of vectors $\widetilde{X}$ using the Cholesky decomposition of the overlap with iterative refinement.

Input: non orthogonal vectors $\widetilde{X}$, threshold τ_{ortho}.
Output: X, orthonormal vectors.

$X = \widetilde{X}$
while $\|X^T X - Id\| > \tau_{\text{ort}}$ **do**
  $M = X^T X$
  Attempt Cholesky factorization $M = LL^T$
  if fail **then**
    Add $\alpha\varepsilon\|X\|$ to the diagonal of M until successful
  end if
  $X = XL^{-T}$
end while

II. ADDITIONAL TESTS FOR THE TD-SCF/DFT CASE

A. Performance and stability on random problems

To further investigate the numerical stability of SMO-GD, we generated random $A+B$ and $A-B$ matrices with an incremental shift on the diagonal equal to two times the index of the column for $A+B$ and one times the index of the column for $A-B$. For this application, the optimal guess was perturbed with random noise in the [0, 0.01] range. The results are reported in Table I.

We reported data only for SMO-GD since it was not possible to converge any tests using the SSF algorithm within the established number of maximum iterations (i.e., 100).

#Eigenvalues	Iterations	Total time (s)
10	34	61.2
20	24	89.3
30	18	109.4
40	17	132.3
50	16	156.2
60	15	179.6
70	15	200.3
80	14	222.9
90	14	245.4
100	14	273.1

TABLE I. Total number of iterations and total time for solving a $2n \times 2n$ problem with $n = 10000$ seeking an incremental number of eigenpairs (from 10 to 100). Convergence threshold is 10^{-6} in the RMS of the residual.

B. Stress test: tight convergence

Using the simple case matrices reported in the main paper and an optimal guess perturbed with random noise, we tested the stability of SMO-GD with regards to tight convergence criteria. In particular, we request a 10^{-10} threshold in the RMS of the residual.

#Eigenvalues	Iterations	Total time (s)
10	14	27.9
20	14	55.3
30	13	79.6
40	13	108.2
50	13	127.2
60	13	158.0
70	13	182.2
80	13	212.7
90	13	230.2
100	13	243.4

TABLE II. Total number of iterations and total time for solving a $2n \times 2n$ generalized problem with $n = 10000$ seeking an incremental number of eigenpairs (from 10 to 100). Convergence threshold is 10^{-10} in the RMS of the residual.

Similarly to the previous tests in subsection II.A, the SSF algorithm was not able to converge any case to the established convergence threshold within 100 iterations. In particular, we note that the RMS of the residual stagnates around $10^{-6} \div 10^{-8}$ depending on the problem.
